# IRF7-Associated Immunophenotypes Have Dichotomous Responses to Virus/Allergen Coexposure and OM-85-Induced Reprogramming

**DOI:** 10.3389/fimmu.2021.699633

**Published:** 2021-07-22

**Authors:** Emma de Jong, Jean-Francois Lauzon-Joset, Jonatan Leffler, Michael Serralha, Alexander N. Larcombe, Claus T. Christophersen, Patrick G. Holt, Deborah H. Strickland, Anthony Bosco

**Affiliations:** ^1^ Telethon Kids Institute, Perth, WA, Australia; ^2^ University of Western Australia, Nedlands, WA, Australia; ^3^ Centre de Recherche, Institut Universitaire de Cardiologie et de Pneumologie de Québec, Université Laval, Quebec, QC, Canada; ^4^ School of Public Health, Curtin University, Perth, WA, Australia; ^5^ WA Human Microbiome Collaboration Centre, School of Molecular and Life Sciences, Curtin University, Bentley, WA, Australia; ^6^ Centre for Integrative Metabolomics and Computational Biology, School of Medical & Health Sciences, Edith Cowan University, Joondalup, WA, Australia

**Keywords:** asthma exacerbation, rhinovirus, allergen, immunomodulation, IRF7, systems biology

## Abstract

High risk for virus-induced asthma exacerbations in children is associated with an IRF7lo immunophenotype, but the underlying mechanisms are unclear. Here, we applied a Systems Biology approach to an animal model comprising rat strains manifesting high (BN) *versus* low susceptibility (PVG) to experimental asthma, induced by virus/allergen coexposure, to elucidate the mechanism(s)-of-action of the high-risk asthma immunophenotype. We also investigated potential risk mitigation *via* pretreatment with the immune training agent OM-85. Virus/allergen coexposure in low-risk PVG rats resulted in rapid and transient airways inflammation alongside IRF7 gene network formation. In contrast, responses in high-risk BN rats were characterized by severe airways eosinophilia and exaggerated proinflammatory responses that failed to resolve, and complete absence of IRF7 gene networks. OM-85 had more profound effects in high-risk BN rats, inducing immune-related gene expression changes in lung at baseline and reducing exaggerated airway inflammatory responses to virus/allergen coexposure. In low-risk PVG rats, OM-85 boosted IRF7 gene networks in the lung but did not alter baseline gene expression or cellular influx. Distinct IRF7-associated asthma risk immunophenotypes have dichotomous responses to virus/allergen coexposure and respond differentially to OM-85 pretreatment. Extrapolating to humans, our findings suggest that the beneficial effects OM-85 pretreatment may preferentially target those in high-risk subgroups.

## Introduction

Rhinovirus infections are important triggers of severe asthma exacerbations among children (1, 2), in particular those who are sensitized to perennial aeroallergens (3). Very few treatment options exist, and drug development programs to redress this deficiency are currently stalled because the underlying immunobiology is incompletely understood (2). To address this issue, we previously employed gene expression profiling to elucidate upper airway responses in children who present to emergency departments with severe exacerbations of asthma or wheeze. We found that the response patterns were heterogeneous and could be divided into IRF7hi *versus* IRF7lo immunophenotypes (4). IRF7hi children were characterized by upregulation of IRF7/antiviral response gene networks, whereas responses in IRF7lo children lacked a corresponding antiviral signature, and instead upregulated genes associated with cytokine and growth factor signaling pathways (e.g. CSF3, EGF, IL-4R, IL-6, IL-10, TGFβ). Moreover, expression of the IRF7lo immunophenotype was associated with more prolonged symptoms during exacerbations and shorter intervals between exacerbation events (4).

In parallel to our studies in children we developed a rat model of respiratory viral infection (5) employing attenuated mengovirus as an alternative to human rhinovirus (6). The approach entailed a comparison of BN and PVG rats, which are respectively characterized by high *versus* low susceptibility to chronic, allergen-driven type 2 (T2)-associated airways inflammation. In a preliminary study focusing on bronchoalveolar lavage (BAL) we found that naïve PVG and BN rats elicit dichotomous responses to infection mirroring to some extent the IRF7hi (PVG) and IRF7lo (BN) phenotypes we observed in children. PVG rats upregulated the expression of type I interferon-related pathways (Mx1, ISG15, IRF7, CXCL10) and lacked markers of T2 inflammation. In contrast, BN rats upregulated markers of T2 inflammation (IL-25, IL-33, ARG1) and lacked a type I interferon signature. Importantly, sensitization of the animals followed by virus/allergen coexposure unleashed a severe airways inflammatory response, which was dominated by eosinophils and orders of magnitude higher in BN *versus* PVG rats even though viral loads were comparable (5). We also investigated the impact of the timing of virus/allergen coexposure on ensuing airways inflammation, and we found that allergen exposure within 24 hours of viral challenge resulted in maximally severe airways inflammation. In the present study we have extended these investigations to encompass comprehensive systems-level analyses of underlying gene coexpression networks in lung and bone marrow tissues, and sought firstly to elucidate the molecular basis for these apparent differences in IRF7-related response profiles following virus/allergen co-exposure in PVG *versus* BN rats, and secondly to test the hypothesis that the asthma risk-associated IRF7lo immunophenotype can be reprogrammed *via* pretreatment with the immune training agent OM-85 to increase resistance to virus/aeroallergen challenge.

## Materials and Methods

### Animals

Brown Norway (BN) and Piebald Virol Glaxo (PVG) rats were bred in-house at the Telethon Kids Institute (Perth, Australia) under specified-pathogen-free conditions, with food and water *ad libitum* and a 12 h light/dark cycle. Eight- to twelve-week-old male rats were utilized for all experiments. All animal experiments were formally approved by the Telethon Kids Institute Animal Ethics Committee, which operates under guidelines developed by the National Health and Medical Research Council of Australia for the care and use of animals in scientific research (Ethic approval number AEC320).

### Viral Model of Experimental Asthma

The model employs BN and PVG rats, previously shown by us to exhibit respectively high *versus* low susceptibility to aeroallergen-induced airways inflammation (7), mimicking the human atopic/non-atopic dichotomy. In follow-up studies the two strains were shown to demonstrate correspondingly disparate levels of susceptibility to severe airways inflammation triggered by respiratory viral infection (5), which in the high-susceptibility BN strain was further amplified by co-exposure to aeroallergen. In the present experiments rats from both strains were sensitized with 0.1mg ovalbumin (OVA)/500 μl Alum 14 days prior to infection with attenuated mengovirus (vMC_0_), followed by aerosol challenge (Ultraneb; DeVilbiss, Somerset, PA, USA) with 1% OVA (Sigma Aldrich, St Louis, MO, USA) for 30 minutes at 1 day-post-infection (D). vMC_0_ was prepared as previously described (6, 8) and rats were inoculated *via* intranasal administration with 100 μl of 10^7^ plaque-forming units (PFU) of vMC_0_ (9). The inflammatory response was assessed at D1, 2 and 9 by bronchoalveolar lavage (BAL) with 8 mL GKN (11mM; glucose, 5.5mM; KCl, 137mM; NaCl, 25mM; Na2HPO4) + 5% FCS. Cell types were identified using Diff-Quick staining of cytocentrifuged samples.

### OM-85 Pretreatment

OM-85 (OM Pharma; Geneva, Switzerland) is an endotoxin-low lyophilized extract containing multiple *Toll-like receptor* (TLR) ligands derived from 8 major bacterial pathogens (*Haemophilus influenzae, Streptococcus pneumoniae, Streptococcus pyogenes, Streptococcus viridans, Klebsiella pneumoniae, Klebsiella ozaenae, Staphylococcus aureus,* and *Neisseria catarrhalis*) frequently associated with respiratory tract infections. OM-85 (single-batch) was administered orally *via* pipette at a dose of 40mg/kg body weight in PBS per day from 7 days after sensitization until infection. This daily dose rate is 10-fold lower than that used in the majority of previous animal model studies carried out by our group and others.

### Single-Cell Suspension Preparation

Single-cell suspensions of lungs and airway draining lymph nodes were prepared by enzymatic digestion as previously described (10). Peripheral blood was harvested by cardiac puncture and mixed with heparin and mononuclear cells (PBMC) were isolated using Histopaque (Sigma-Aldrich) gradient enrichment per the manufacturer’s instructions. Cells were resuspended in PBS with 0.1% Bovine Serum Albumin (BSA). Bone marrow cells (BM) were harvested from tibia bones *via* flushing with 10 ml GKN + 5% FCS as previously described (11).

### Flow Cytometry

Single-cell suspensions of lungs, lymph node, PBMC and BM were used for immunostaining using CD45, RT1b, CD3, CD161, CD11b, CD4, CD8, CD127, CD25, CD43, CD172a, CD278, CD45R (BD Pharmingen, San Jose, CA, USA; eBiosciences, San Diego, CA, USA; BioLegend, San Diego, CA, USA; Miltenyi Biotec, Bergisch‐Gladbach, Germany; Bioss, Woburn, MA, USA; Abcam, Cambridge, UK; R&D Systems, Minneapolis, MN, USA). Intracellular FoxP3 staining was conducted using the eBiosciences FoxP3 intracellular staining buffer set. Data were collected on a four-laser LSRFortessa (BD Biosciences) and analyzed using FlowJo software (Version 10.0.7, Tree Star, Sanford, CA, USA). See supplementary **Figure S1** in the Online Repository for complete gating strategy.

### RNA Isolation and Sequencing

BM and lung tissue were resuspended in RNAlater (Ambion, Life Technologies, Mulgrave, VIC, Australia) and frozen at -80°C. Total RNA was extracted with TRIzol (Ambion) followed by RNAeasy (Qiagen Gmbh, Hilden, Germany). Total RNA libraries were prepared using TruSeq Stranded mRNA Library Prep Kit (Illumina Inc, San Diego, CA) and sequenced by the Australian Genome Research Facility (Illumina HiSeq2500, 50bp single-end reads, v4 chemistry). On average, ~46 million reads were generated per sample. The raw sequencing data are available from GEO (GSE157441).

### RNA-Seq Data Analysis

Read libraries were quality assessed using FastQC (12) (v0.11.3) and mapped to the rat genome (rn6) using HISAT2 (13) (v2.0.4). Gene-level quantitation (counts) of aligned reads was performed using SummerizeOverlaps (14), and post-alignment quality control was performed using SAMStat (15) (v1.5.2). All downstream analyses unless otherwise specified were performed in the R environment for statistical computing (v3.5.1). Sample QC was performed by examining relative log expression, density plots and principal component analysis, and one outlier (with low RNA quality) was removed from further analyses. Differentially expressed genes were identified between experimental groups (within tissues) using DESeq2 (16), following minimal gene filtering to remove genes with a summarized count of zero or one. Molecular drivers of the differentially expressed genes were identified using upstream regulator analysis (Ingenuity Systems) (17). Results were filtered to remove chemicals and drugs, to focus on biological mechanisms. Activation Z-scores were calculated for each molecular driver by comparing their known effect on downstream targets with observed changes in gene expression; activation Z-scores ≥ 2 or ≤ -2 were considered activated or inhibited, respectively. P-values derived from DESeq2 and upstream regulator analysis were adjusted for multiple comparisons using the Benjamini-Hochberg method; those <0.05 were considered significant. The weighted gene co-expression network analysis (WGCNA) (18) algorithm was used to construct signed networks for lung and bone marrow samples separately, with further separation by animal strain; resulting in four distinct networks. To prepare data for WGCNA, we (i) applied a variance stabilizing transformation to normalize the data, (ii) filtered out genes expressed at a low level (only those with at least 10 counts per sample were retained), (iii) removed genes without an official Rat Genome Database symbol and (iv) removed genes with low variability by applying the varianceBasedfilter() function within the Differentially Coexpressed Gene/Link (DCGL) package (19) (significance threshold set to 0.05). The Gene Ontology Consortium database was used to identify over-represented biological processes and molecular functions associated with the differentially expressed genes and network modules, using Fisher’s exact tests and Bonferroni-corrected p-values; those <0.05 were considered significant (20, 21). Protein-protein interaction networks were constructed for network modules of interest using mouse orthologs [sourced from the Mouse Genome Database (22)] and Network Analyst (v3.0) (23). Here, the STRING interaction database (v10) (22) was used with default settings for other parameters. Network type was set to “minimum” to focus on key connectivities and reduce the number of first-order neighbours.

### Microbiome Analysis

Fecal pellets were collected fresh for each rat (weighing on average 200 mg) and then stored at -80°C. DNA was extracted using the QIAamp PowerFecal DNA kit (Qiagen, Hilden, Germany) with the addition of mechanical lysis for 2 x 30s on a TissueLyser II (Qiagen, Hilden, Germany). Extractions were automated using a QIAcube Connect (Qiagen, Hilden, Germany) following the manufacturer’s instructions. DNA concentrations were estimated using the QIAxpert System (Qiagen, Hilden, Germany). PCR inhibitors were assessed by a three-point dilution of each sample prior to the amplicon PCR using quantitative polymerase chain reaction (qPCR) with identical cycling conditions and primers as for amplicon PCR. The amplicon PCR was performed using the highest concentration of DNA showing no presence of inhibitors for each sample with the V4 primers (515F and 806R) labelled with unique dual barcodes for each sample, followed by PCR-free ligation of sequence adapters. The MiSeq sequencing set-up was carried out as per the manufacturers protocol. The amplicon library was bidirectionally sequenced using a 500-cycle V2 reagent kit and a V2 Standard flow cell (Illumina, USA). Sequence read quality was initially assessed with FastQC before demultiplexing and preprocessing by GHAPv2, an in-house tool. Cutadapt (24) was used for removal of all non-biological sequences before DADA (25) was used for quality filtering, error correction, amplicon sequence variants (ASVs) picking. For this study the forward sequences were trimmed at 180 bp and the reverse at 155 bp to maintain a constant read quality of >30 and the overlapping region between the two reads were >80 bp. A trained naïve bayes classifier (RDP) (26) then assign nomenclature to ASVs against the curated Genome Taxonomy Database (27). Following assignment, decontamination was performed with decontam (28) removing sequences not present in minimum 4 samples with a minimum of 10 reads.

### Statistical Analysis

Statistical analyses were performed using GraphPad Prism software (V8, La Jolla California USA). Details are described within figure legends; P-values <0.05 were considered significant. Microbial analysis was conducted using Primer7 with Permanova^+^ (PRIMER-e, Quest Research Limited, NZ). At the phylum and genus level, relative abundance data were square root transformed, prior to the calculation of a Bray-Curtis dissimilarity matrix. Principal coordinates analysis was used to examine possible differences or separations among the groups visually at the 3-D level. This was followed by multivariate analysis using PERMANOVA^+^ to statistically assess differences between groups and changes over time. The model used for this analysis was a 3-factor model, with group (PVG or BN), rats ID (nested within group) and time point. Test of individual phyla or genera differences between groups were also done using PERMANOVA^+^.

### Study Approval

All experiments were performed under guidelines from the National Health and Medical Research Council of Australia and approval of the institutional Animal Ethics Committee.

## Results

### Sensitized PVG and BN Rats Exhibit Dichotomous Responses to Virus/Allergen Coexposure

**Figure 1:**
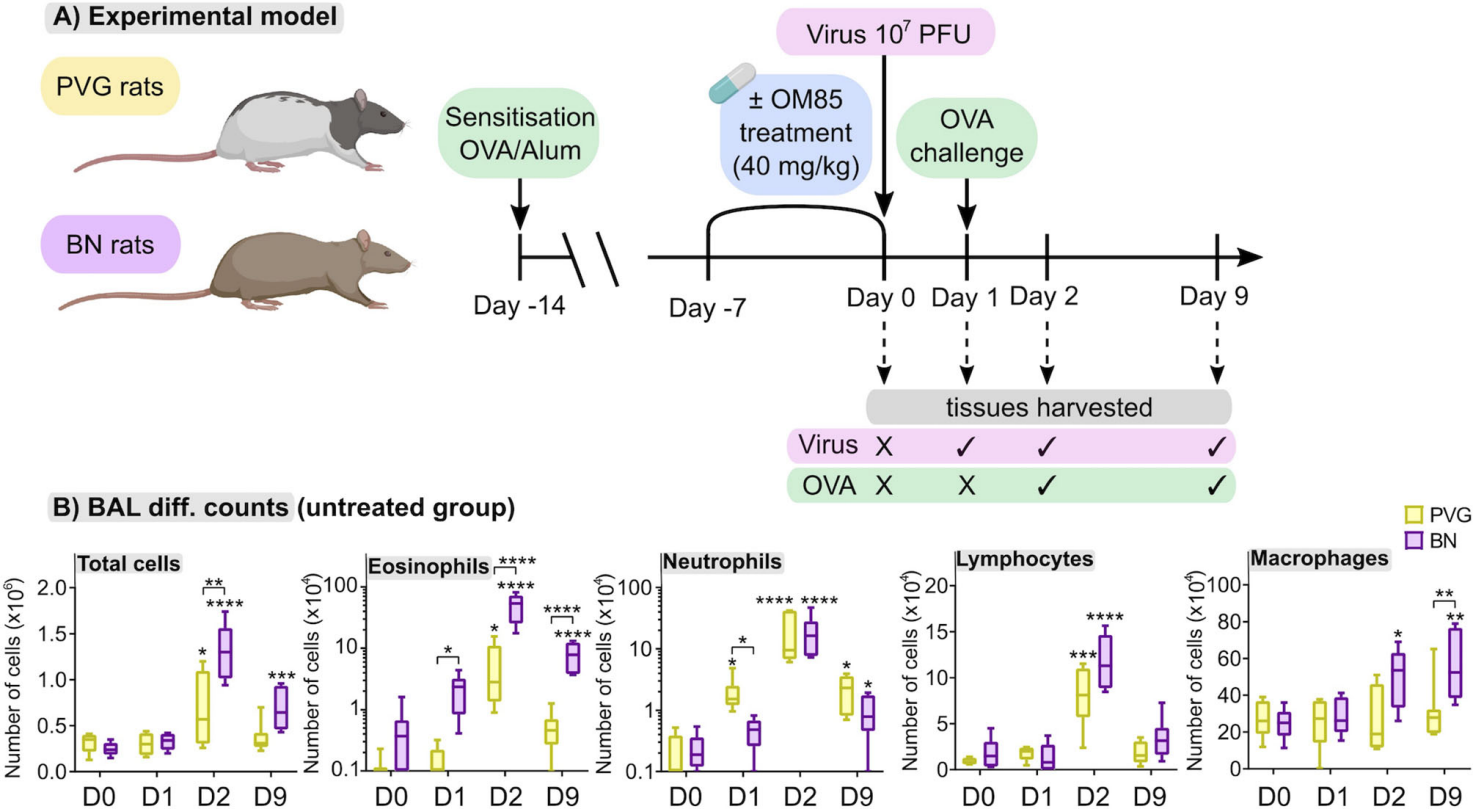
PVG and BN rats exhibit distinct cellular profiles of airways inflammation following virus/allergen coexposure. **(A)** PVG and BN rats were sensitized with OVA/Alum. Seven days later, rats from each strain were randomly allocated to experimental groups receiving OM-85 for seven consecutive days or nil treatment. All animals apart from a baseline control group (Day 0) were then infected with attenuated mengovirus and challenged with OVA 24hr later. Tissues were harvested at baseline, one day post-infection representing the response to virus alone, and days two and nine post-infection representing the response to virus and allergen co-exposure. The analyses performed included i) differential cell counts on BAL fluid, ii) RNA-Seq on homogenized lung and bone marrow, iii) 14-colour flow cytometry on single-cell suspensions of PBMC, airway draining lymph node, bone marrow and peripheral lung tissue, and iv) assessment of the gut microbiome (prior to virus/allergen coexposure only). Figure created with BioRender.com **(B)** Enumeration of total cells, eosinophils, neutrophils, lymphocytes, and macrophages in BAL *via* differential cell counts at baseline and following viral/OVA challenge in PVG and BN rats without OM-85 treatment. Data from more than 3 independent experiments, n = 6–8 per group. Two-way ANOVA with Tukey’s multiple comparisons test were performed to compare data within and between strains. *p < 0.05, **p < 0.01, ***p < 0.001, and ****p < 0.0001. BAL, bronchoalveolar lavage, D0: day 0, D1: day 1, D2: day 2, D9: day 9.

We first aimed to characterize responses to virus/aeroallergen coexposure in PVG and BN rats across time through a comprehensive systems biology approach. **Figure 1A** presents an overview of the model and all analyses performed. Assessment of airways inflammation highlighted two key features that distinguish PVG from BN rats. First, the acute (day 1; D1) inflammatory response to virus in PVG rats was dominated by neutrophil influx in BAL, in contrast to the acute eosinophilic response observed in BN rats (**Figure 1B**). Neutrophil recruitment was however observed in BN rats following virus/allergen coexposure, to levels consistent with PVG rats peaking at day 2 (D2). Second, BN rats exhibited marked eosinophilia at all time points, while PVG rats displayed transient eosinophilia. Importantly, BAL eosinophilia in BN rats remained significantly elevated at day 9 (D9), demonstrating incomplete resolution of T2 inflammation.

### PVG and BN Rats Mobilize Distinct Gene Network Patterns in the Lung and Bone Marrow

**Figure 2:**
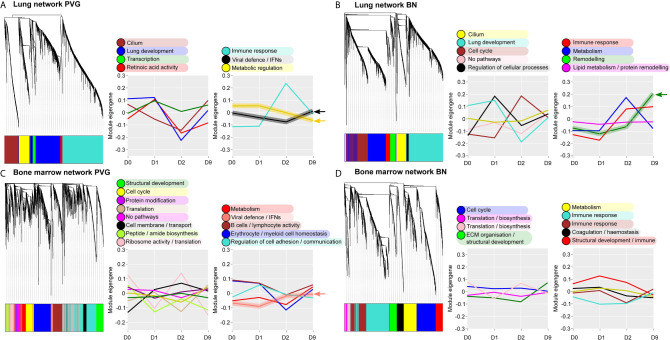
Overview of the global gene networks operating in lung and bone marrow of PVG and BN rats. Separate tissue-specific weighted co-expression gene networks were generated for PVG and BN lung **(A, B)** and bone marrow **(C, D)**. Dendrograms are shown for each network, where distinct color blocks represent separate functional modules (clusters of correlated or co-expressed genes). Associated module functions are described for each network and the summarized expression of each module (eigengene value) is shown over the course of viral infection and allergen challenge. Modules relating to immune and metabolic pathways are separated (right) from all others (left). Arrows highlight four modules specific to PVG or BN rats. Sample sizes for network construction: BN bone marrow n = 47, PVG bone marrow n = 49, BN lung n = 48, PVG lung n = 47.

**Figure 3:**
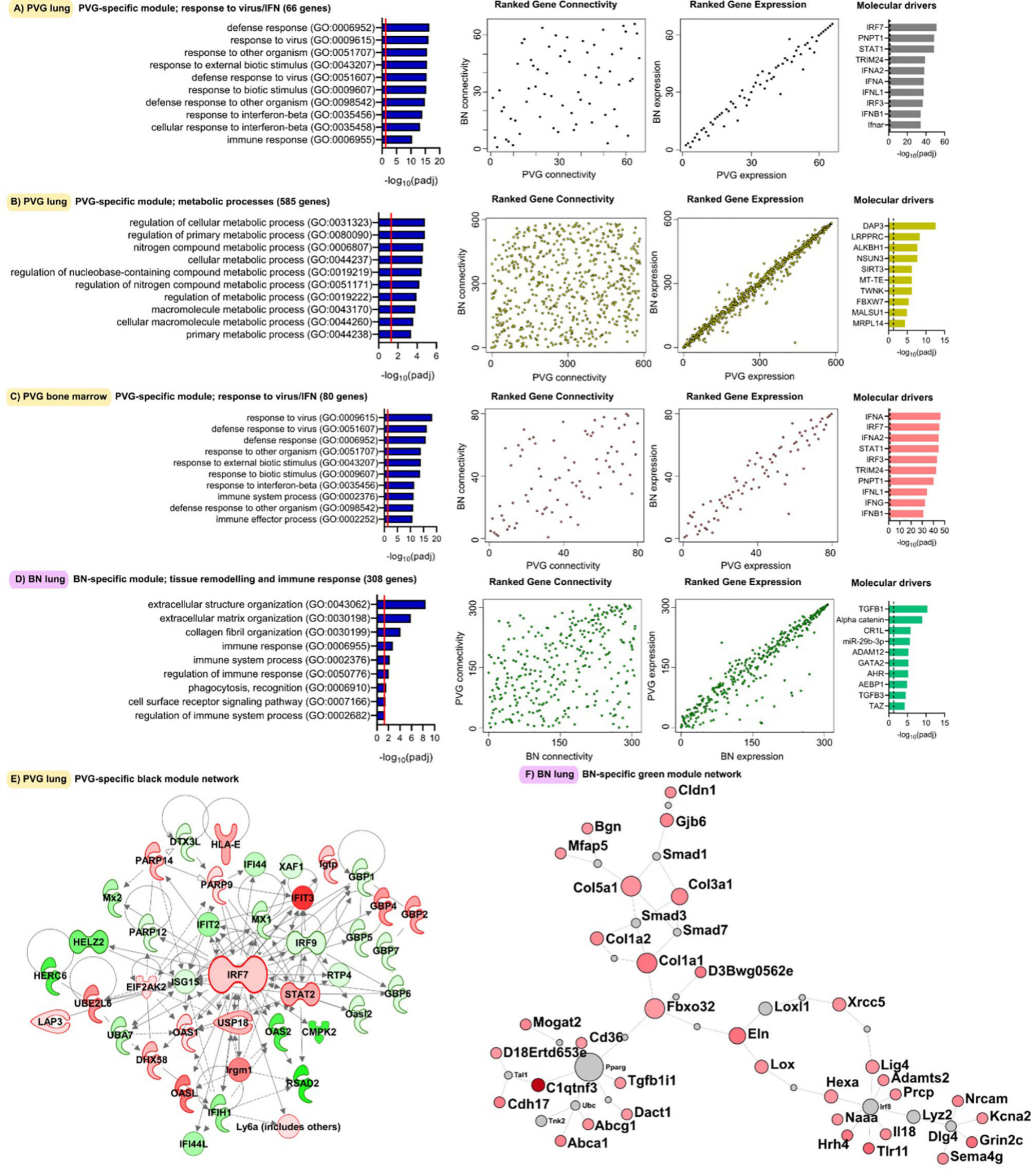
Functional gene networks unique to either PVG or BN rats. The four gene network modules unique to either PVG lung **(A, B)**, PVG bone marrow **(C)** or BN lung **(D)**. Over-represented biological pathways are shown for each module, the red line at 1.3 represents the threshold for significance equivalent to a Bonferroni-corrected p-value <0.05. For each network module, ranks of gene connectivity (reflecting the relative importance of that gene within the network module) and gene expression were calculated for both PVG and BN: correlation plots between rat strains are shown. The top ten predicted molecular drivers are shown for each module, where the dashed line represented the threshold for significance. Prior knowledge network reconstructions are shown for the PVG lung black module **(E)** and the BN lung green module **(F)** where red and green coloring indicate increased and decreased expression respectively on day 2 (for **E**) and day 9 (for **F**) relative to day 0. Sample sizes for network construction: BN bone marrow n = 47, PVG bone marrow n = 49, BN lung n = 48, PVG lung n = 47.

Next, we analyzed global patterns of gene expression in the lung to provide a deeper understanding of responses to virus/allergen coexposure. Given accumulating evidence that atopic asthma exacerbations are in part mediated through a lung/bone marrow axis (29), we analyzed gene expression patterns in bone marrow in parallel. We generated tissue-specific weighted co-expression gene networks for PVG and BN rats to reconstruct the global architecture and functional organization of the gene expression program occurring within each strain. Using this approach, we identified seven modules in PVG lung, and nine in BN lung, and pathways analysis demonstrated that the modules were enriched for specific biological functions (**Figures 2A, B**). To determine if the modules were preserved between strains, we calculated module preservation statistics, and compared ranked expression and ranked connectivity patterns in PVG *versus* BN (**Table S1** and **Figure 3**). We found that two PVG-specific lung modules had markedly different connectivity patterns in BN rats: an interferon/antiviral response module and a metabolic regulation module. Reconstruction of the interferon/antiviral response module using experimentally supported molecular interactions from the Ingenuity Systems KnowledgeBase in combination with Upstream Regulator Analysis demonstrated that *Irf7* is predicted to function as a hub and key driver of this module (**Figures 3A, E**). Similar reconstruction of the metabolic regulation module using InnateDB revealed several members of the circadian clock as hub genes (*Clock*, *Per1*, *Per3*; **Figure S2** in the Online Repository), and core regulators of mitochondrial homeostasis among the top predicted molecular drivers (*Dap3*, *Lrpprc*, *Nsun3*, *Sirt3*; **Figure 3B**). We also identified one BN lung module associated with airways remodeling with markedly different connectivity patterns in PVG, the expression of which increased with coexposure to virus/allergen and peaked at day nine post-infection (**Figure 2B**). Transforming growth factor beta 1(*Tgfb1*) was the top predicted driver for this module (**Figure 3D**), and several collagen genes were among the top hubs (*Col1a1*, *Col3a1*, *Col1a2*, *Col5a1*; **Figure 3F**). We next examined network topology heatmaps for the three lung modules of interest, and found that genes from the PVG-specific *Irf7*/antiviral and metabolic regulation modules failed to form cohesive networks in BN lung (**Figure S3**). Conversely, the BN-specific lung remodeling/*Tgfb1* module did not form a cohesive unit in PVG rats.

WGCNA analysis of gene expression patterns in bone marrow cells revealed thirteen functional modules in PVG rats, and nine in BN rats (**Figures 2C, D**). Again, we identified one PVG-specific network module associated with *Irf7*/antiviral responses (**Figures 3C**, **S4** in the Online Repository). As observed in lung tissue, the network connectivity patterns for this *Irf7* module in PVG bone marrow module were discordant between BN and PVG rats (**Figures 3C**, **S3** in the Online Repository). All other bone marrow modules were preserved between strains (**Table S2** in the Online Repository). In summary, our findings demonstrate that cohesive *Irf7*/antiviral gene networks are present in PVG lung and bone marrow but are absent from these tissues in BN rats. In contrast, only BN rats exhibit a dedicated lung network module associated with remodeling/*Tgfb1* signaling, and peak expression of this module was observed on day 9. This further supports that PVG mirrors IRF7hi responders, whereas BN resemble IRF7lo responders.

### Virus/Allergen Coexposure Induces Exaggerated and Sustained Transcriptional Changes in the Lungs of BN Rats

**Figure 4:**
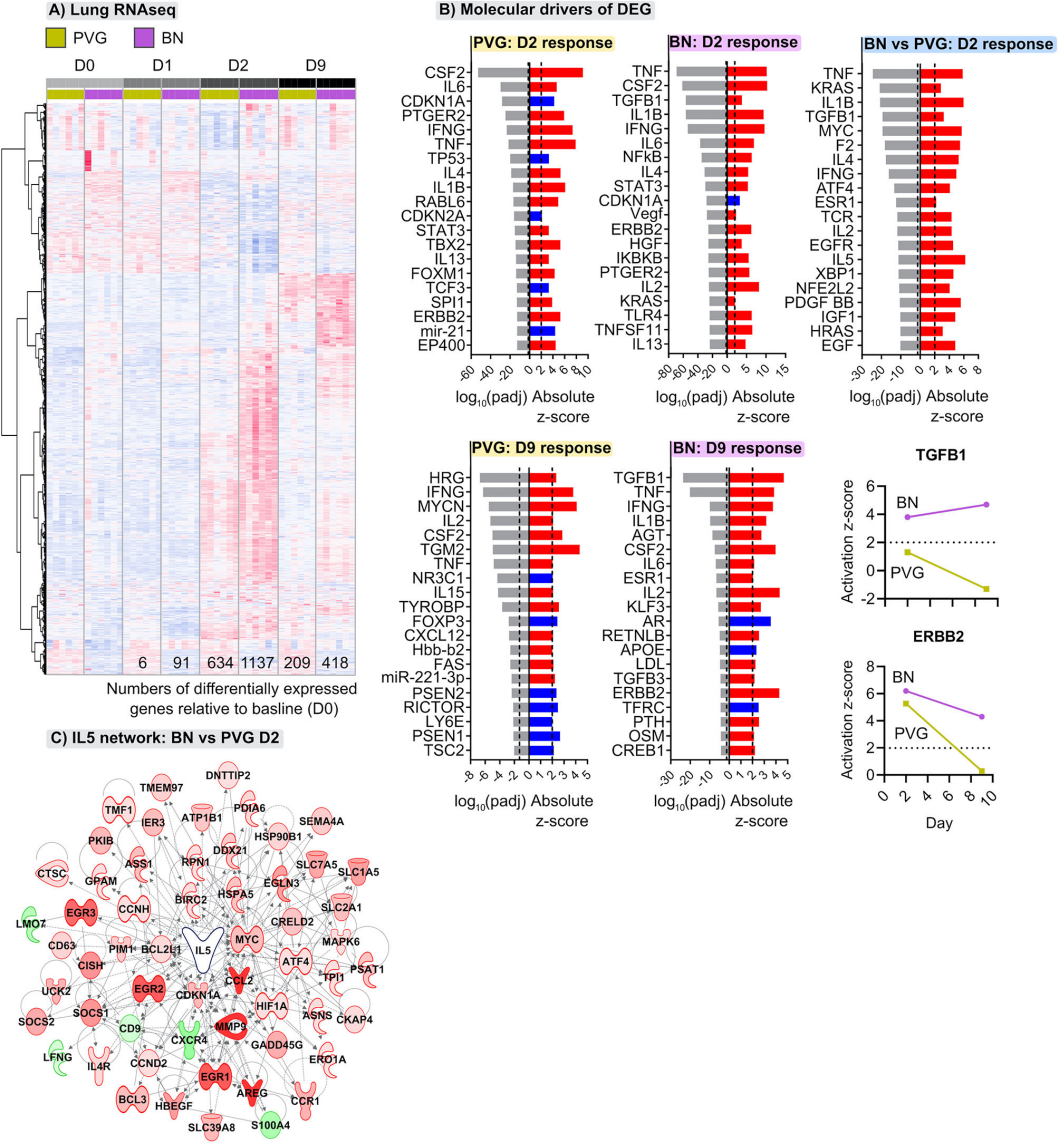
Distinct transcriptional changes occur in lungs of PVG and BN rats following viral- and allergen-exposure. **(A)** Heatmap visualizing the union of differentially expressed genes induced following viral infection and OVA challenge within either PVG or BN rats; genes ranked using unsupervised hierarchical clustering. Red and blue coloring indicate relative increased or decreased expression for each gene, respectively. Data from more than 3 independent experiments with n=6 per time point. **(B)** The top twenty predicted upstream transcriptional regulators of the differentially expressed genes at day two and nine. Red and blue indicate predicted activation (Z-score > 2) and inhibition (Z-score < -2), respectively. P-values were adjusted for multiple comparisons using the Benjamini-Hochberg method; the dashed line at -1.3 represents the threshold for significance. Activation Z-scores for TGFB1 and ERBB2 are compared between strains over the course of infection and allergen challenge. **(C)** Network wiring diagram for IL5 where nodes are colored based on comparison between BN and PVG day 2 responses; red and green indicate increased and decreased expression in BN relative to PVG on day 2, respectively.

The above analyses provide a global view of the functional organization and connectivity structure of the gene expression program in PVG and BN in the context of virus/allergen coexposure. We next aimed to identify sets of genes that were differentially expressed over time in the respective responses and identify underlying molecular drivers. The greatest transcriptional changes were observed in the lungs of both strains following virus/allergen coexposure on day 2 (**Figure 4A**). However, the BN-response was much more intense than that of PVG rats, even at day 9 post-infection. Employing Upstream Regulator Analysis (17) we demonstrated that the peak response to virus/allergen coexposure in both strains was mediated by canonical drivers of inflammation (*Tnf*, *Il1b*, *Ifng*, *Il6*) and T2 pathways (*Il4, Il5*), but that these pathways were exaggerated in BN rats (**Figures 4A, B**). Indeed, *Il5* was the top ranked regulator in terms of activation Z-score, and the expression of most genes downstream of *Il5* was elevated in BN relative to PVG on day 2 (**Figure 4C**). In addition, activation of growth factor signaling (*Tgfb1*, *Erbb2*, *Vegf*, *Hgf*) was a distinct feature of the BN lung response, with *Tgfb1* still the predominant driver at day nine (**Figure 4B**). Interestingly, analysis of bone marrow revealed limited changes in gene expression in BN (<7 differentially expressed genes across all comparisons), and more than 200 differentially expressed genes in PVG (79 and 153 differentially expressed genes at day one and nine post-infection, respectively; **Tables S3**, **S4** in the Online Repository). The day nine changes in PVG bone marrow were associated with decreased expression of genes involved in ribosomal activity, and increased expression of genes involved in metabolic pathways (**Figure S5** in the Online Repository).

### PVG and BN Rats Display Differential Tissue Recruitment of Innate Lymphoid Cells, Dendritic Cells and Neutrophils Following Virus and Allergen Exposure

**Figure 5:**
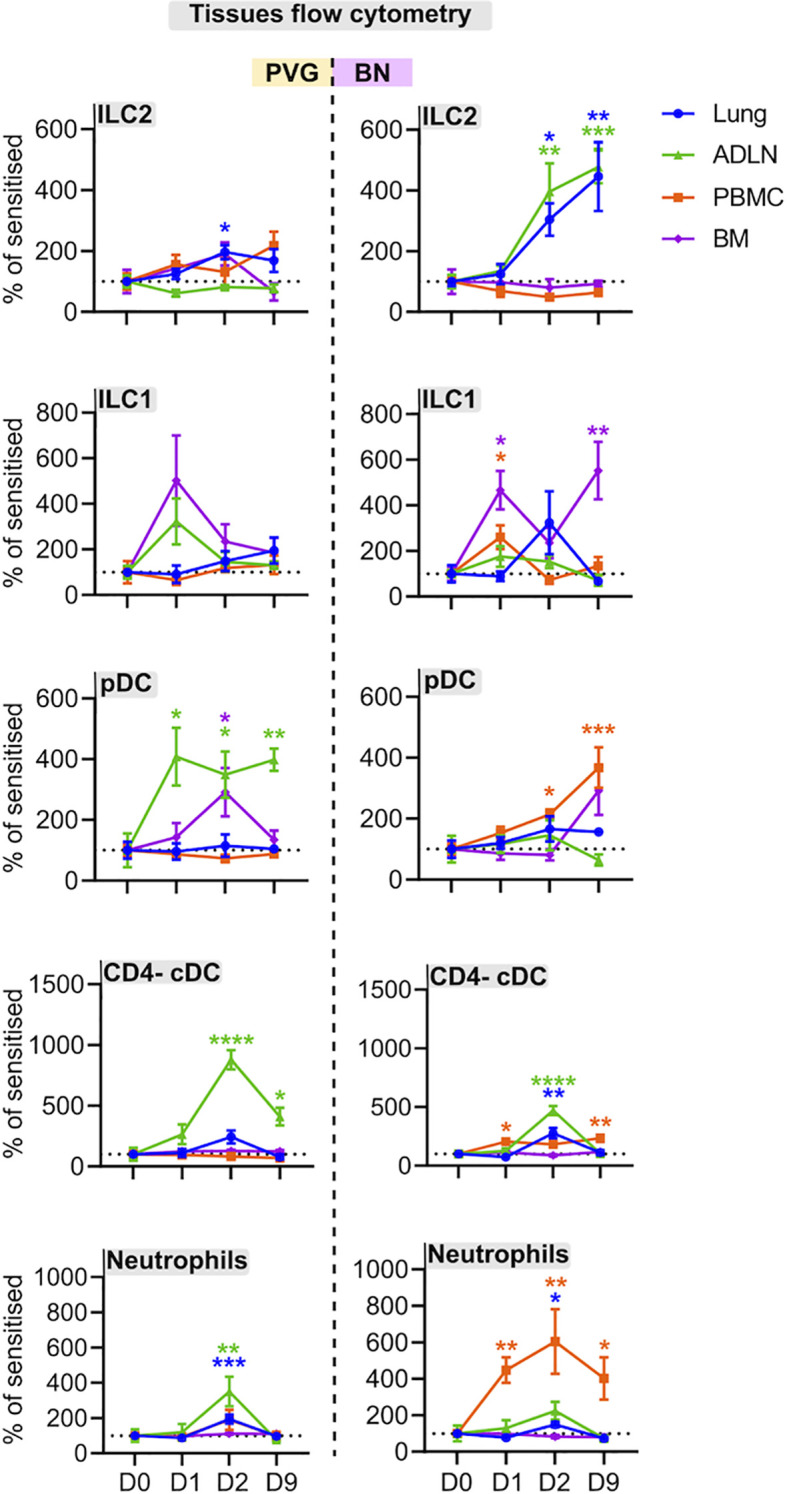
PVG and BN rats display differential cellular recruitment into tissues following challenge with virus and allergen. Flow cytometric analysis of lung, bone marrow, airway draining lymph node (ADLN), and PBMC over the course of viral infection and allergen challenge. Data are presented as the percentage of CD45+ cells (mean ± standard error of the mean) normalized to Day 0, from more than 3 independent experiments with n = 4-6 per strain, tissue, and time point. One-way ANOVA with Dunnett’s multiple comparisons test was performed to determine differences at each time point relative to baseline, within PVG and BN separately. *p < 0.05, **p < 0.01, ***p < 0.001, and ****p < 0.0001.

We employed flow cytometry to examine local and systemic changes in immune cell populations (**Figure S1**). The PVG response to virus/allergen coexposure was characterized by early peaks in plasmacytoid dendritic cell (pDC) influx in the airway draining lymph node, and transient increases in type 2 innate lymphoid cells (ILC2), neutrophils and CD4- conventional dendritic cells (cDCs) which all peaked at day two post-infection in the airway draining lymph node and/or lung (**Figures 5**, **S6**). In contrast, the BN response was skewed towards expansion of ILC populations, where ILC2 frequencies in the lung and airway draining lymph node progressively increased to reach their highest levels on day nine post-infection, concurrent with elevated bone marrow ILC1 cells (**Figures 5**, **S6**). Further, BN rats displayed delayed pDC recruitment, and a sustained increase in neutrophils within the periphery that was not observed in PVG rats.

### Sensitized PVG and BN Rats Exhibit Differences in Cellular and Molecular Profiles at Baseline

**Figure 6:**
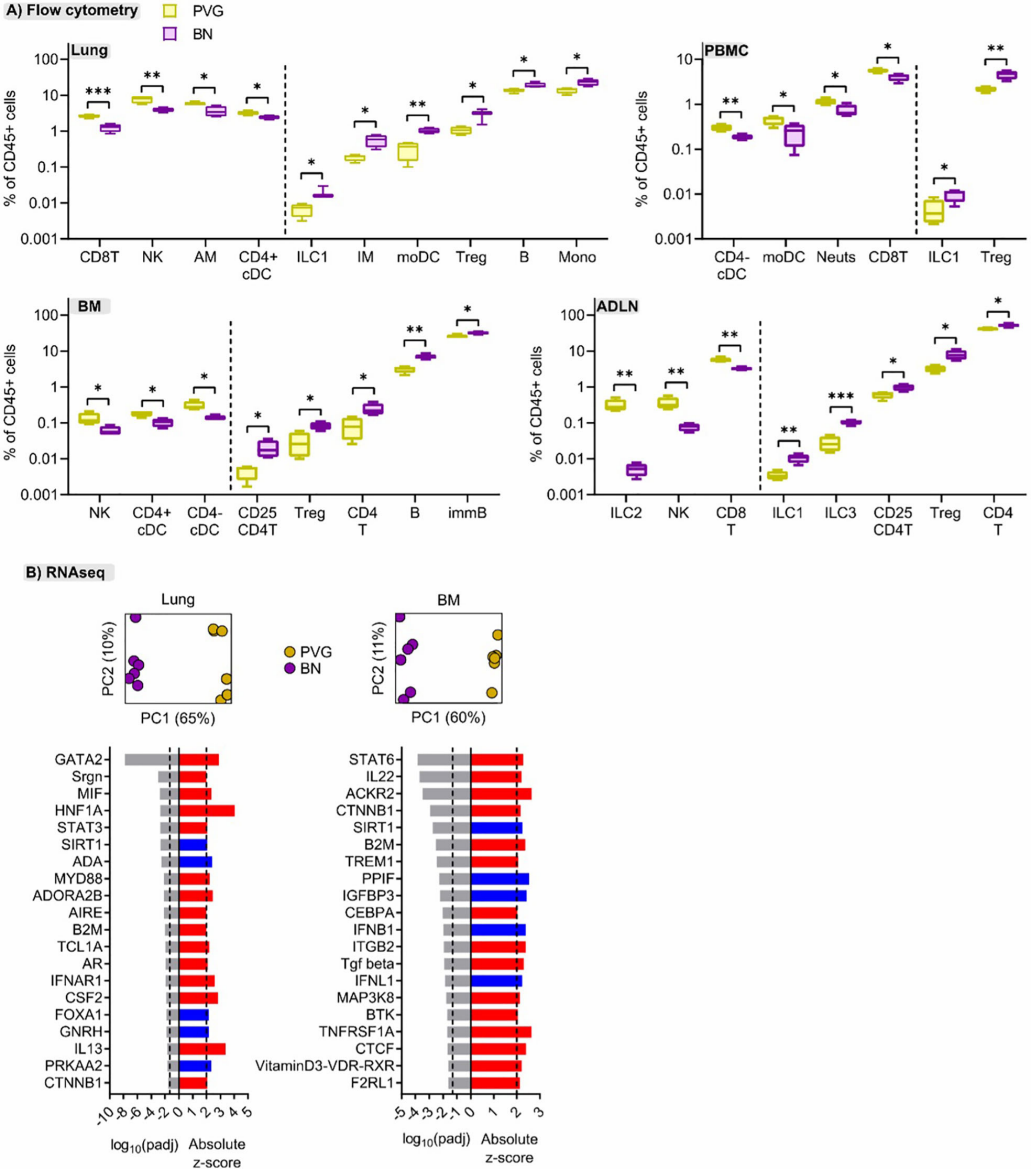
Cellular and molecular differences are evident between sensitized PVG and BN rats. **(A)** Flow cytometric analysis of lung, bone marrow (BM), airway draining lymph node (ADLN), and PBMC revealed differences in the proportions of several immune cell types at baseline (day 0, untreated) between strains. Data from more than 2 independent experiments with n = 4 per strain, T-tests comparing PVG (yellow) and BN (purple) rats were performed for each cell type within each tissue; *p < 0.05, **p < 0.01, and ***p < 0.001. **(B)** Principal component analysis using the top 1000 variable genes across samples revealed distinct separation between PVG and BN rats, in both tissues (Data from more than 2 independent experiments with n = 6 each). The top twenty predicted upstream transcriptional regulators of the differentially expressed genes between PVG and BN rats are presented. Red and blue indicate predicted activation (Z-score > 2) and inhibition (Z-score < -2), respectively. P-values were adjusted for multiple comparisons using the Benjamini-Hochberg method. The dashed line at -1.3 represents the threshold for significance.

To better understand the mechanisms contributing to the divergent responses to virus/allergen coexposure between PVG and BN rats, we compared their cellular and molecular profiles across tissues at baseline. Overall, PVG rats had higher proportions of natural killer cells (NK) and CD8 T cells in multiple tissues, mirrored by decreased proportions of T-regulatory cells (**Figure 6A**). In BN rats, ILC populations were elevated compared to PVG and skewed towards ILC1/3, particularly in the lymph nodes. Stark differences between strains were also evident at the molecular level. Principal component analysis revealed distinct gene expression profiles between PVG and BN rats at baseline in both lung and bone marrow (**Figure 6B**), where 60–65% of transcriptional variation was attributed to strain alone. Differential gene expression analysis identified 1078, and 924 genes with an absolute fold-change in expression >2 between PVG and BN rats in the lung and bone marrow, respectively (**Tables S5**, **S6** in the Online Repository). Genes with increased expression in BN rats were enriched for several immune response pathways in both lung and bone marrow (**Figure S7A** in the Online Repository). Interestingly, we also observed a relative dampening of viral-defense pathways in the bone marrow of BN rats (**Figure S7B** in the Online Repository). These differences were predicted to be driven by activation of several molecules relating to maintenance/activation of myeloid cells in the lung (*Gata2*, *Mif*, *MyD88*, *Csf2*), alongside the hallmark T2 cytokine *Il13* (**Figure 6B**). In contrast, the differences in bone marrow profiles were characterized by activation of *Stat6* and *Trem1* signaling and inhibition of viral response pathway regulators (*Ifnb1* and *Ifnl1*, **Figure 6B**).

### OM-85 Pretreatment Has Distinct Effects on PVG and BN Responses to Virus and Allergen

**Figure 7:**
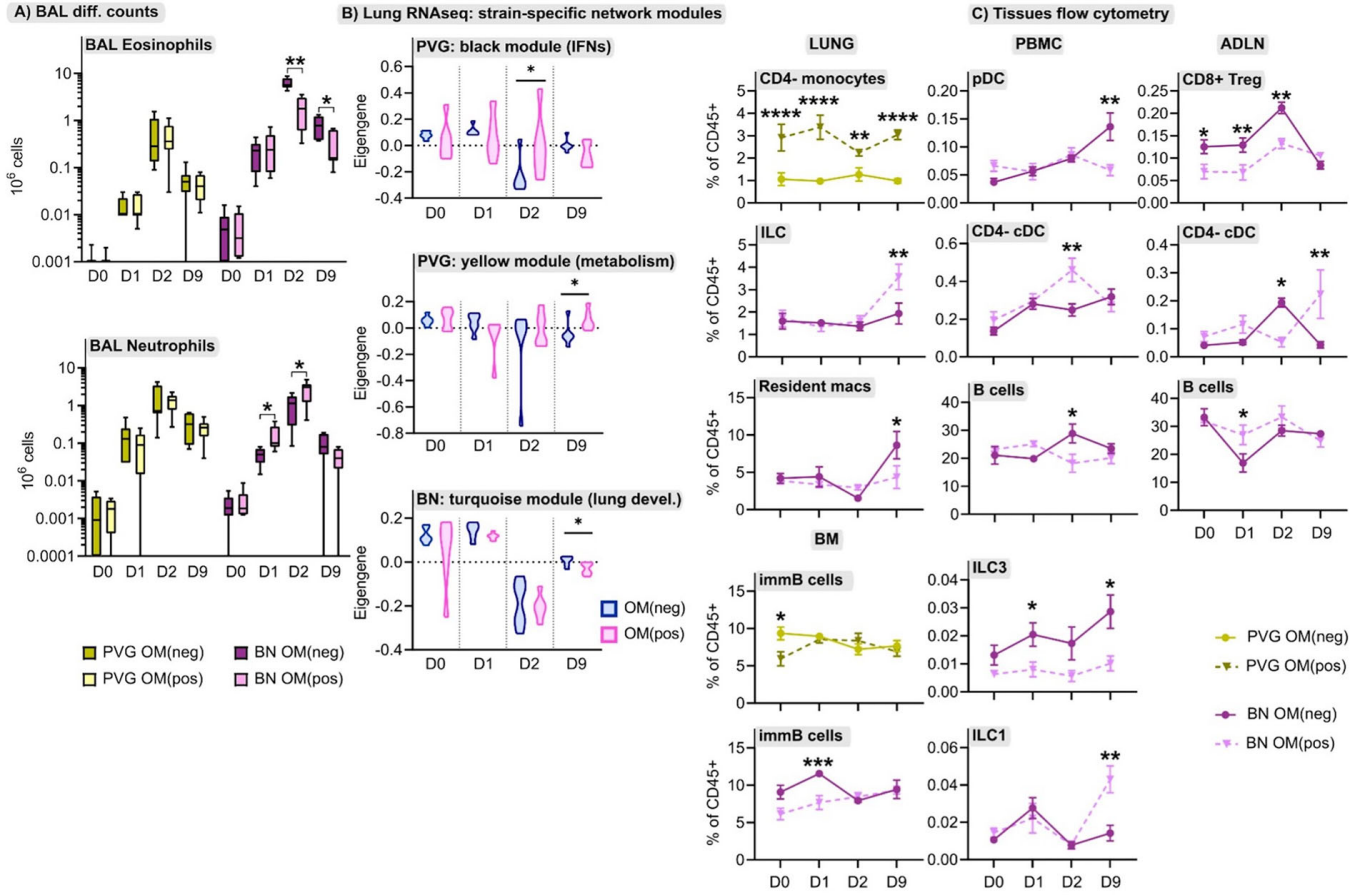
The effect of OM-85 on responses to virus and allergen. **(A)** Enumeration of eosinophils and neutrophils in BAL *via* differential cell counts at baseline, and following the course of viral infection and allergen challenge in PVG and BN rats with and without OM-85 pretreatment. Data from more than 3 independent experiments with n = 5-6 per strain, treatment group and time point. **(B)** The effect of OM-85 pretreatment on gene networks within lung tissue, presented as violin plots of summarized gene expression (eigengene values). Data from more than 3 independent experiments with n = 6 per strain and treatment group. **(C)** Flow cytometric analysis of lung, PBMC, airway draining lymph node (ADLN) and bone marrow over the course of viral infection and allergen challenge with and without OM-85 treatment. Data are presented as mean ± standard error of the mean, from more than 3 independent experiments with n = 3-7. Statistical comparisons between OM(pos) and OM(neg) samples were performed using one-way ANOVA with Sidak’s multiple comparisons tests in **(A)** and **(C)**, and Mann-Whitney tests in **(B)**. *p < 0.05, **p < 0.01, ***p < 0.001, and ****p < 0.0001.

The immunomodulator OM-85 shows promise as a safe and effective preventative treatment for protection against airways inflammation triggered by a diverse range of stimuli (30), but has not been studied in the context of IRF7-associated immunophenotypic responses to virus/allergen coexposure. Here, we evaluated OM-85 pretreatment as a potential therapeutic strategy to attenuate airways inflammation in PVG and BN rats. The data showed that OM-85 had no impact on neutrophil or eosinophil influx in PVG rats over the time-course of virus/aeroallergen coexposure (**Figure 7A**). In BN rats however, OM-85 significantly boosted the acute neutrophilic response to virus on day 1, concomitant with a reduction of eosinophilia on days 2 and 9 (**Figure 7A**). Consistent with the BAL data, OM-85 pretreatment was associated with significant gene expression changes in baseline lung tissue from BN rats (178 differentially expressed genes; **Table S7** in the Online Repository) but had close to no effect on PVG lung tissue (only 2 differentially expressed genes). The most significant over-represented biological processes associated with OM-85-mediated molecular changes in BN lung were associated with innate and adaptive immunity, in particular increased expression of *Egr1*, *Cxcl9*, and *Il12rb1*, which play key roles in antiviral defense (**Tables S7**, **S8**). In contrast, assessment of OM-85 pretreatment effects in bone marrow on the basis of global gene expression revealed only minor changes in both PVG and BN rats. As a complimentary analysis, we looked at the effect of OM-85 on the expression of our defined network modules in lung and bone marrow. Here, OM-85 pretreatment was associated with increased eigengene values (representing summarized gene expression) for the PVG lung *Irf7*/antiviral and metabolic regulation modules (day two and nine post-infection, respectively), and reduced expression of the BN module related to lung development at day nine post-infection (**Figure 7B**). We also examined the effect on OM-85 on the expression of selected IRF7 pathway genes in whole lung, and although the findings were mostly not significant, there was a trend for stronger effects of OM-85 in BN compared to PVG particular at D2 post infection (**Figure S8**). OM-85 pretreatment had no significant impact on the expression of bone marrow network modules in either strain. Finally, we observed cellular changes associated with OM-85 in all tissues, predominantly in BN rats (**Figure 7C**). These changes included alterations to the B cell compartment in PBMC, airway draining lymph node and bone marrow, and shifts in DC/ILC populations in PBMC, lung and lymph node.

### PVG and BN Rats Have Distinct Gut Microbiome Profiles Which Are Modified by OM-85 Pretreatment

**Figure 8:**
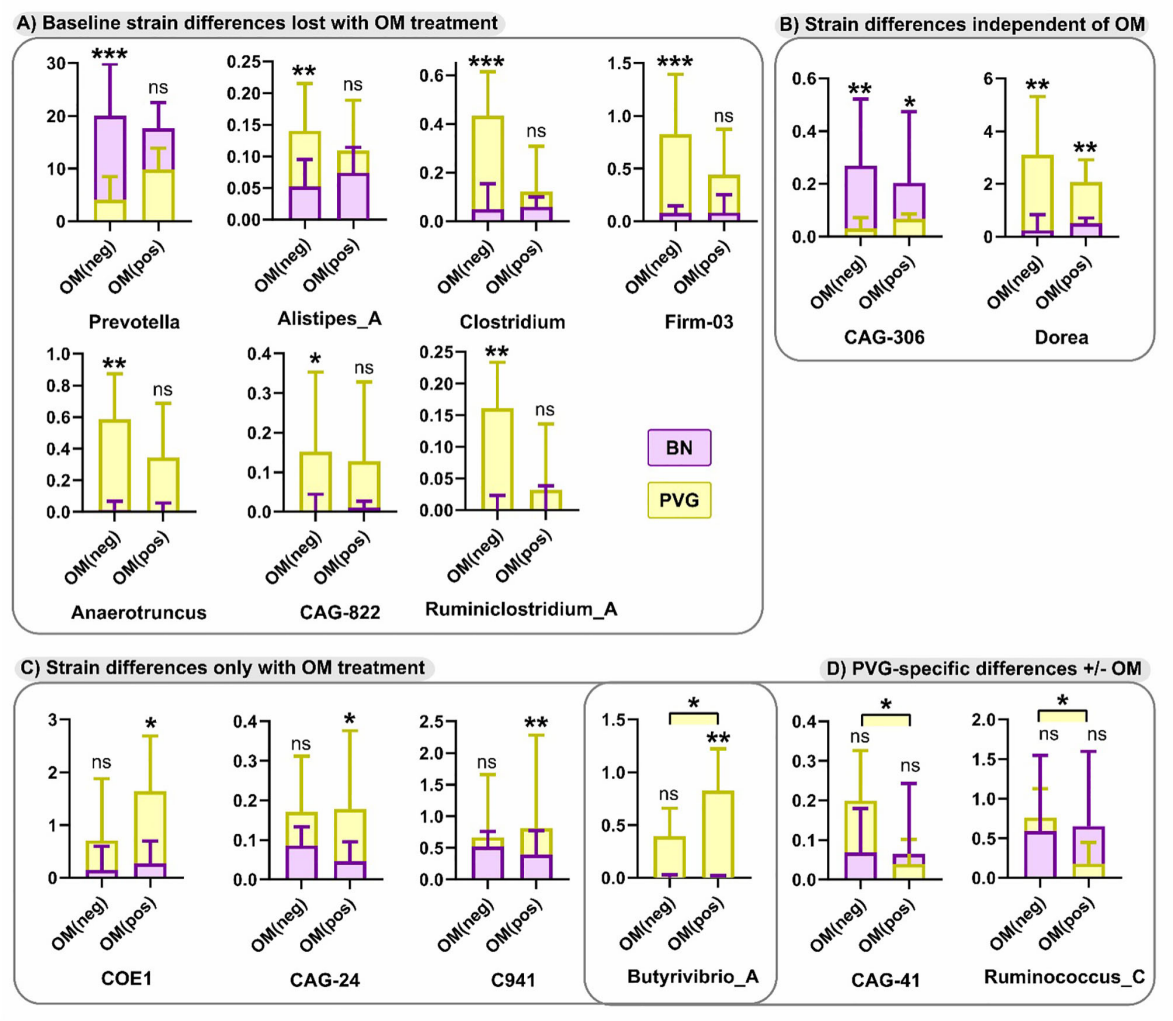
Differences in the microbiome between PVG and BN rats at with and without OM-85 treatment. The effect of OM-85-pretreatment on the microbiome at genus-level. Observed differences were either; evident at baseline (Day 0) without treatment, but no longer different between strains with OM-85 treatment **(A)**, evident between strains independent of OM-85 treatment **(B)**, only evident between BN and PVG rats with OM-85 treatment **(C)**, or evident within PVG rats when comparing treatment groups **(D)**. Results are presented as relative abundance; bars show median and upper quartile from more than 3 independent experiments with n = 12 per group. NS, not significant; *p < 0.05, **p < 0.01 and ***p < 0.001.

Finally, in view of the growing evidence of a role for the microbiome in regulating airways inflammation (31), we conducted an exploratory analysis of potential effects of OM-85 preventative treatment on respective gut microbiome profiles in the two strains. At the phylum level pre-treatment, both strains were dominated by Bacteroidetes and Firmicutes (**Table S9** in the Online Repository), however the relative abundance of Bacteroidetes was significantly higher in BN rats (mean ± SEM of 69 ± 3% *vs* 56 ± 4%, p=0.0108). In addition, the overall composition of the microbiota at the genus level was different between strains (p=0.0467), including a higher relative abundance of Prevotella in BN rats, paralleled by a lower relative abundance of Anaerotruncus and Dorea (**Figures 8A, B**). Assessment of the microbiome in droppings following OM-85 pretreatment showed an overall convergence between PVG and BN rats: several genera that were different at baseline between strains including Prevotella, were similar post-treatment (**Figure 8A**). Further, statistical assessment of the differences between PVG and BN rats at the genus-level was no longer significant (p=0.105). There were, however, some new differences in specific genera post-OM-85 preventative treatment including Butyrivibrio (**Figure 8C**), while Dorea remained significantly more abundant in PVG rats regardless of OM-85 treatment (**Figure 8B**). Within-strain analysis identified three genera significantly altered in PVG rats only following OM-85 pretreatment, while no treatment-associated changes were specific to BN rats (**Figure 8D**).

## Discussion

Inflammatory response profiles amongst children during severe asthma exacerbations are characterized by IRF7hi and IRF7lo immunophenotypes; the latter phenotype is associated with prolonged symptoms and shorter intervals between exacerbation events, however the immunological and molecular features that underpin these dichotomous profiles are not well understood (4). The sub-group of children at highest risk of severe exacerbations are atopics who are sensitized to perennial (in particular indoor) aeroallergens (1, 2, 29, 32). Concomitant exposure to the latter during respiratory viral infections is virtually unavoidable. To gain new insight into the contribution of variations in IRF7-associated immunophenotypes in this context, we utilized an experimental rat model of virus/allergen co-exposure using two strains that mirror these dichotomous IRF7 phenotypes and characterized each employing a comprehensive systems biology approach. IRF7 gene networks were readily identified in the lung and bone marrow of PVG rats following exposure, yet were completely absent from these compartments in BN rats. Moreover, we observed divergent patterns of airways inflammation between strains. Early neutrophil recruitment in BAL and plasmacytoid dendritic cells in airway draining lymph nodes were unique to PVG rats, while BN rats displayed persistent eosinophilic inflammation in BAL concomitant with progressive recruitment of ILC2 cells in the lung/airway. Further, BN rats displayed exaggerated airways inflammation through upregulation of proinflammatory responses (e.g. TNF, IL-1B, IFNG, IL-4, IL-5), and genes involved in TGFβ signaling and airways remodeling. Importantly, we also observed differences between PVG and BN rats in terms of their response to immunomodulating therapy with OM-85. In PVG rats, OM-85 pretreatment boosted the overall expression of lung IRF7 gene networks but did not alter BAL airways inflammation. In contrast, OM-85 pretreatment increased neutrophilic airways inflammation to infection and decreased eosinophilic responses to virus/allergen coexposure in BN. Finally, microbiome profiles at baseline were distinct in PVG and BN rats, and this was mitigated in part by OM-85 preventative treatment. In summary, we have identified the immunological and molecular hallmarks that underpin IRF7 phenotypes in an experimental rat model, and further demonstrated that these phenotypes respond differentially to OM-85 pretreatment. Our findings have implications for the design of translational studies that employ microbial products to reprogram innate immune responses for the prevention of virus-induced exacerbation of allergic airways disease.

Upper airway gene expression profiling in both children and adults with asthma demonstrate heterogenous interferon responses during *in vivo* rhinovirus infection (4, 33), where dampened or dysregulated expression of interferon genes is inversely correlated with severity of clinical symptoms and viral clearance. Here, we have used PVG and BN rats to model these distinct immunophenotypes, allowing investigation beyond the upper airway. Our previous comparisons between PVG and BN rats in the setting of viral infection and/or allergen exposure have focused on local immune responses in the airway and associated mucosa (5, 7). Consistent with those studies, we report rapid neutrophil recruitment to the airway alongside moderate and self-resolving eosinophilia following challenge with virus and allergen in sensitised PVG rats. Extending our previous work to comprehensive molecular characterisation of lung tissue we identified a small (<100 genes) yet highly specialised gene network associated with interferon signaling in PVG rats, with IRF7 at its core. IRF7 is a transcription factor critical for type I interferon production in response to viral infection, downstream of viral-engagement with various pattern recognition receptors (34), and is a major hub connecting molecular networks that are induced in the respiratory tract during viral-induced asthma exacerbations (35). The importance of IRF7-dependent interferon production in anti-viral immunity has been extensively validated, and is perhaps best exemplified by human and animal studies in which genetic IRF7 deficiencies are associated with heightened susceptibility to severe (respiratory) viral infections (36, 37). Moreover, PVG rats displayed rapid expansion of pDCs in the airway draining lymph nodes following viral infection. These cells are the most potent producers of type I interferon, exhibit high constitutive expression of IRF7 (38), and are responsible for 70–80% of the gene expression program induced by human rhinovirus in PBMC (39). We observed conservation of the IRF7 gene network in the bone marrow of PVG rats, and increased frequencies of bone marrow pDCs two days post-viral infection, supporting an active role for the bone marrow during direct environmental insults to the airway. Remarkably, neither pDC mobilisation in the airway or bone marrow, or formation of IRF7 gene networks in these compartments were apparent in BN rats. While BN rats are not IRF7 deficient, they exhibit a complete breakdown in the underlying molecular wiring of the IRF7 gene networks that in contrast, form cohesively in PVG rats, suggesting dysregulation of this pathway in BN rats. Failure to orchestrate a rapid immune response *via* IRF7 following viral infection in a T2hi biased system may also contribute to exacerbated airways inflammation and remodeling, given the inverse relationship between IRF7 and IL-33 expression (key driver of type 2 inflammation) observed in human airway cells *in vitro* (40) and *ex vivo* (4), and in animal models (41). In this regard it is pertinent to note that while expression of the human T2hi immunophenotype confers markedly increased risk of atopic asthma as illustrated by epidemiological findings demonstrating that whilst >80% of asthmatic teenagers are atopic, <20% of teenagers sensitized to aeroallergens actually develop clinically significant airways disease, suggesting that additional risk factor(s) must operate in synergy with atopy to precipitate disease expression (42), and it is tempting to speculate that the IRF7lo immunophenotype may play such a role.

Indeed, the response dynamics unique to BN rats reflect a scenario of on-going eosinophilia and exacerbated airways inflammation driven by growth factors, TGFβ1 in particular. TGFβ1 signaling is mediated intracellularly *via* the phosphorylation and subsequent nuclear translocation of SMAD2/3 transcription factors (43), and both genome-wide association and epigenetic studies have identified associations between components of the TGFβ1 signalling pathway and asthma risk (44, 45). Further, TGFβ1 was identified as a putative driver of the molecular changes specific to IRF7lo viral asthma exacerbations (4), and recent transcriptomic analyses of nasal epithelium confirms a SMAD3-centered molecular signature associated with both viral and non-viral asthma exacerbations in children (46). We also identified a gene network specific to BN lung that was associated with airways remodeling, in which several collagen genes were among the most highly interconnected hubs. Aberrant collagen deposition is characteristic of airway remodelling in asthma (47), and recent evidence directly implicates eosinophils in the expression of collagen in airway smooth muscle *via* TGFβ1 signalling in humans with allergic asthma (48), providing a circuit connecting the features distinct to BN rats in our model. In addition to the central role of TGFβ in airways remodeling (49), this multifunctional cytokine is required for the generation and functional maintenance of ILC2 cells (50), which were progressively recruited to the lung and airway draining lymph nodes in BN rats but only transiently (and moderately) elevated in lungs of PVG rats. ILC2 cells promote T2 inflammation and eosinophilia in the airway (51), again showcasing the high-allergen-responder phenotype of BN rats. Finally, a molecular signature associated with increased T2 signalling (driven by STAT6, a mediator of IL-4/IL-13 signaling) and dampened interferon was evident in the bone marrow of BN rats at baseline, mirrored by reduced proportions of cDCs and increased CD4 T cells compared with PVG. Collectively, these findings demonstrate an imbalance between interferons and T2 signalling between sensitised PVG and BN rats over the course of viral infection provoked by allergen exposure and links the immunological landscape of the bone marrow to airway vulnerability in our model. Similar immunophenotypes distinguished by the relative strength of T2 and type I interferon signaling are evident in airway mucosa of children with acute viral bronchiolitis (52), and are even predictive of exacerbation risk in upper airway samples (46) highlighting the clinical relevance of these observations.

Importantly, the differences we observed between PVG and BN rats extended beyond their cellular and molecular profiles, to include their response to OM-85 pretreatment. The rationale for employing microbial products to attenuate asthma exacerbations is derived from comparisons of children raised in traditional farming environments, with similar genetic backgrounds, lifestyles, and diets, but strikingly different rates of allergic sensitisation and asthma (53). Notably, comparisons between Amish and Hutterite farm children by Stein et al. identified IRF7 and TNF as key network hubs connecting differentially expressed genes in peripheral blood cells between these populations, both of which were over-expressed in the Amish (i.e. children with low rates of allergies/asthma and high microbial exposures) (53). We did not observe up-regulation of TNF (or associated innate pathways) in either BN or PVG rats following OM-85 pretreatment. As TNF production is triggered by LPS engagement with TLR4 (54), this discrepancy is likely explained by the fact that OM-85 is an endotoxin low product (55), whereas dust from Amish homes contained levels of endotoxin ~7 times higher than in Hutterite homes (53). In our model, OM-85 pretreatment significantly shifted the profile of airways inflammation in BN rats through dampening eosinophilia and expression of gene networks involved in airways remodelling, while boosting neutrophil recruitment. In contrast, OM-85 pretreatment had no effect on the cellular composition of BAL in PVG rats but did enhance overall expression of IRF7 and metabolic gene networks in PVG lung. The contrasting effects of OM-85 pretreatment in PVG *versus* BN rats suggests that the protective effects of microbial products [as observed in children raised in farming environments (56, 57)] may be influenced by genetic background. The mechanism of action of OM-85 we observed in this study is also consistent with findings in other infection models in which OM-85 pretreatment attenuated LPS-induced inflammation (systemic and local) while preserving interferon response pathways (58).

Interestingly, we also detected a molecular signature associated with OM-85 pretreatment in BN lung at baseline, that was not identified in PVG rats. This signature was associated with activation of complement, B-cells, immunoglobulin activity and phagocytosis, in line with early studies on the immunomodulatory properties of OM-85 (59–62), and a more recent viral-challenge animal model demonstrating activation of splenic B cells and production of anti-viral antibodies (in serum and BAL) following oral administration of OM-85 (63). The proposed mechanisms of action of oral OM-85 include activation of immune cells in gut- and mucosal-associated lymphoid tissue (64) and in bone marrow (65), thus comparisons of these tissues between PVG and BN rats post treatment would be of interest in future studies. The fact that OM-85 pretreatment had a more profound effect in BN rats broadly reflects the findings of a recent phase IV clinical trial, where the greatest benefit was seen in children most vulnerable to respiratory tract infections (66).

Another potential mechanism by which OM-85 pretreatment exerts effects on the immune response to virus and allergen is through modulation of the gut microbiome, yet no studies to date have investigated this issue. Here, we report an overall convergence in gut microbiome profiles between PVG and BN rats following OM-85 pretreatment, whereas baseline analyses revealed significantly higher relative abundances of Bacteriodetes (including Prevotella) in BN rats and enrichment of Firmicutes in PVG, echoing human nasal microbiome profiles of subjects with exacerbated, and non-exacerbated asthma, respectively (67). Counterintuitively, all of the statistically significant OM-85-induced differences we observed occurred in PVG rats, although the biological relevance of these changes is uncertain, given the median magnitude of change was roughly half a percent (or less) in terms of relative abundance. Furthermore, other genera were not affected by OM-85 pretreatment: Dorea remained under-represented in T2hi BN rats both pre- and post-OM-85, consistent with other reports of reduced Dorea in stools of children with mite-sensitised rhinitis (68), and young adults with food allergy and sensitisation (69). Our observation that orally administered OM-85 had significant effects on the lung compartment in asthma-susceptible BN rats does suggest that interactions between the gut and lung contribute to disease expression and resolution. Indeed, Arrieta et al. identified four bacterial genera that were associated with asthma risk in infants, and confirmed a causative role for these taxa in ameliorating airways inflammation in an animal model (70). However, whether compositional shifts in the gut microbiome following oral OM-85 are causative of downstream effects or merely associative, remains to be determined.

We acknowledge that our study has limitations. First, molecular assessment of lung and bone marrow was performed through bulk RNA-Seq profiling, thus the levels of gene expression represent the average across multiple different cell populations within each tissue, obscuring the relative contributions of individual cell populations. This limitation is partially overcome through comprehensive cellular profiling of each tissue by flow cytometry, and future studies based on single-cell RNA-Seq technologies will provide a deeper understanding of IRF7 immunophenotypes at single cell resolution. Second, we have focused on characterising the molecular and cellular hallmarks that underpin IRF7 immunophenotypes and their response to OM-85 pretreatment to inform clinical and translational studies, however, detailed mechanistic studies will be required to dissect the precise molecular mechanisms that control these phenotypes, including the specific function of IRF7 in the lung *versus* bone marrow. Notwithstanding these limitations, our findings demonstrate that the IRF7lo immunophenotype markedly increases risk for severe airways inflammation to virus/allergen coexposure, and moreover demonstrate that pretreatment with OM-85 has potential to mitigate susceptibility to ensuing airways inflammation by upregulation of T1-associated pathways (Il12rb1, Cxcl9) and attenuation of T2 inflammation.

## Data Availability Statement

The datasets presented in this study can be found in online repositories. The names of the repository/repositories and accession number(s) can be found below:

https://www.ncbi.nlm.nih.gov/geo/, GSE157441.

## Ethics Statement

All animal experiments were formally approved by the Telethon Kids Institute Animal Ethics Committee, which operates under guidelines developed by the National Health and Medical Research Council of Australia for the care and use of animals in scientific research.

## Author Contributions 

AL, DS, and AB designed and supervised the study. J-FL-J, JL, MS, and CC performed the experiments. EJ, J-FL-J, CC, PH, DS, and AB interpreted the data. EJ, J-FL-J, AL, CC, PH, DS, and AB wrote and revised the manuscript. All authors contributed to the article and approved the submitted version.

## Funding

This study was supported by NHMRC 1129996. JFLJ was supported by a fellowship from the Fonds de recherche du Québec en Santé.

## Conflict of Interest

The authors declare that the research was conducted in the absence of any commercial or financial relationships that could be construed as a potential conflict of interest.
