# Supplementary material for: IRF7-Associated Immunophenotypes Have Dichotomous Responses to Virus/Allergen Coexposure and OM-85-Induced Reprogramming
Source: Front Immunol. 2021 Jul 22;12:699633. doi: 10.3389/fimmu.2021.699633 (PMC8339879; doi:10.3389/fimmu.2021.699633)
Supplement: Supplementary file 2 [file DataSheet_2.zip › Supplementary_figures.pdf]

Supplementary Materials

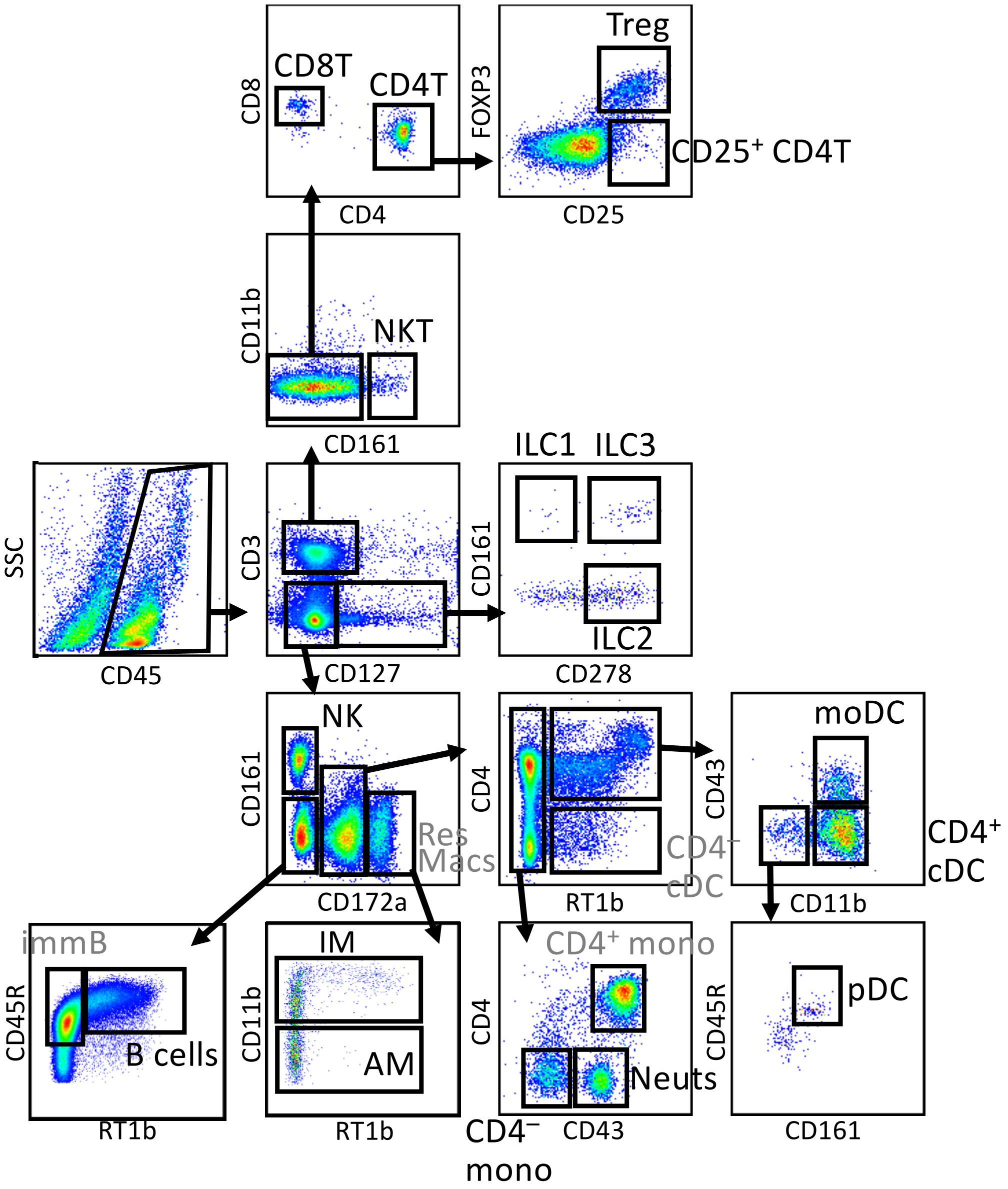

Figure S1. Flow cytometry gating strategy.

Representative strategy used to identify immune subsets (CD45+) within lungs, lymph node, PBMC and bone marrow. Lymphoid cell subsets included: CD4 T cells ($CD3^{+}CD4^{+}CD8^{-}$), CD8 T cells ($CD3^{+}CD4^{-}CD8^{+}$), CD4 Tregs ($CD3^{+}CD4^{+}CD8^{-}CD25^{+}FoxP3^{+}$), CD4 Teff ($CD3^{+}CD4^{+}CD8^{-}CD25^{+}FoxP3^{-}$), NKT cells ($CD3^{+}CD161^{+}$), NK cells ($CD3^{-}CD161^{+}CD172a^{-}$), ILC1 ($CD3^{-}CD127^{+}CD161^{+}CD278^{-}$), ILC2 ($CD3^{-}CD127^{+}CD161^{-}CD278^{+}$) and ILC3 ($CD3^{-}CD127^{+}CD161^{+}CD278^{+}$), and immature B cells (immB; $CD3^{-}CD161^{-}CD172a^{-}CD45R^{+}RT1b^{-}$) and B cells ($CD3^{-}CD161^{-}CD172a^{-}CD45R^{+}RT1b^{+}$). Myeloid cell subsets included: monocyte-derived DC (moDC; $CD3^{-}CD161^{-}CD172a^{+}RT1b^{+}CD4^{+}CD11b^{+}CD43^{+}$), CD4+ cDC ($CD3^{-}CD161^{-}CD172a^{+}RT1b^{+}CD4^{+}CD11b^{+}CD43^{-}$), CD4- cDC ($CD3^{-}CD161^{-}CD172a^{+}RT1b^{+}CD4^{-}$), pDC ($CD3^{-}CD161^{-}CD172a^{+}RT1b^{+}CD4^{+}CD11b^{-}CD43^{-}CD45R^{+}CD161^{+}$), neutrophils (Neuts; $CD3^{-}CD161^{-}CD172a^{+}RT1b^{-}CD4^{-}$ $CD43^{+}$), CD4+ monocytes (CD4+ mono; $CD3^{-}CD161^{-}CD172a^{+}RT1b^{-}CD4^{+}CD43^{+}$) and CD4- mono ($CD3^{-}CD161^{-}CD172a^{+}RT1b^{-}CD4^{-}CD43^{-}$). Lung resident macrophages subsets (ResMacs; $CD3^{-}CD161^{-}CD172a^{high}$) were divided into alveolar macrophages (AM; $CD3^{-}CD161^{-}CD172a^{high}CD11b^{-}$) and interstitial macrophages (IM; $CD3^{-}CD161^{-}CD172a^{high}CD11b^{+}$).

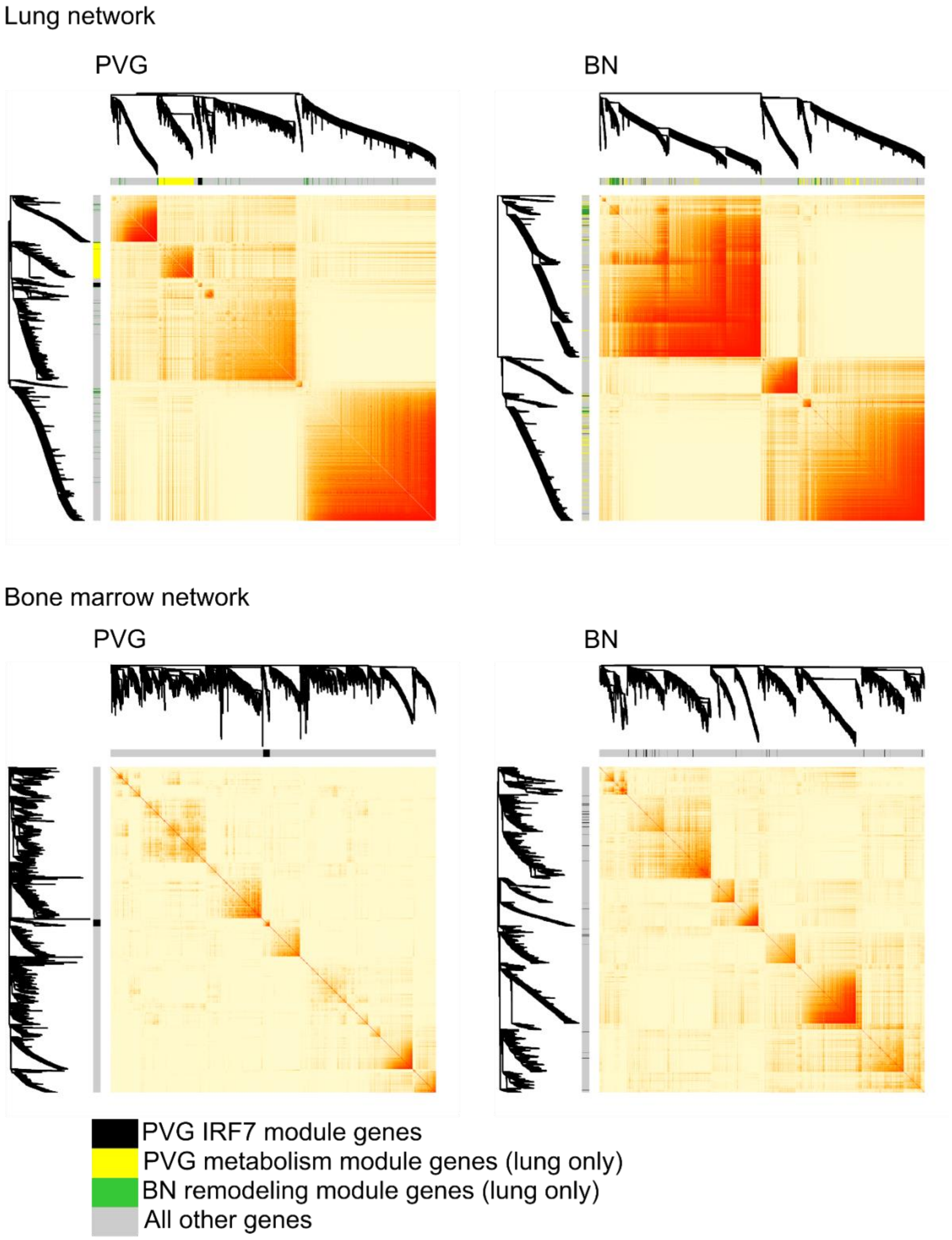

Figure S2. Network patterns of strain-specific modules. The location of genes of interest from strain-specific modules were plotted over a heatmap of the network topology for each strain demonstrating that the PVG-specific modules are dispersed in BN, and in contrast, the BN-specific module is dispersed in PVG.

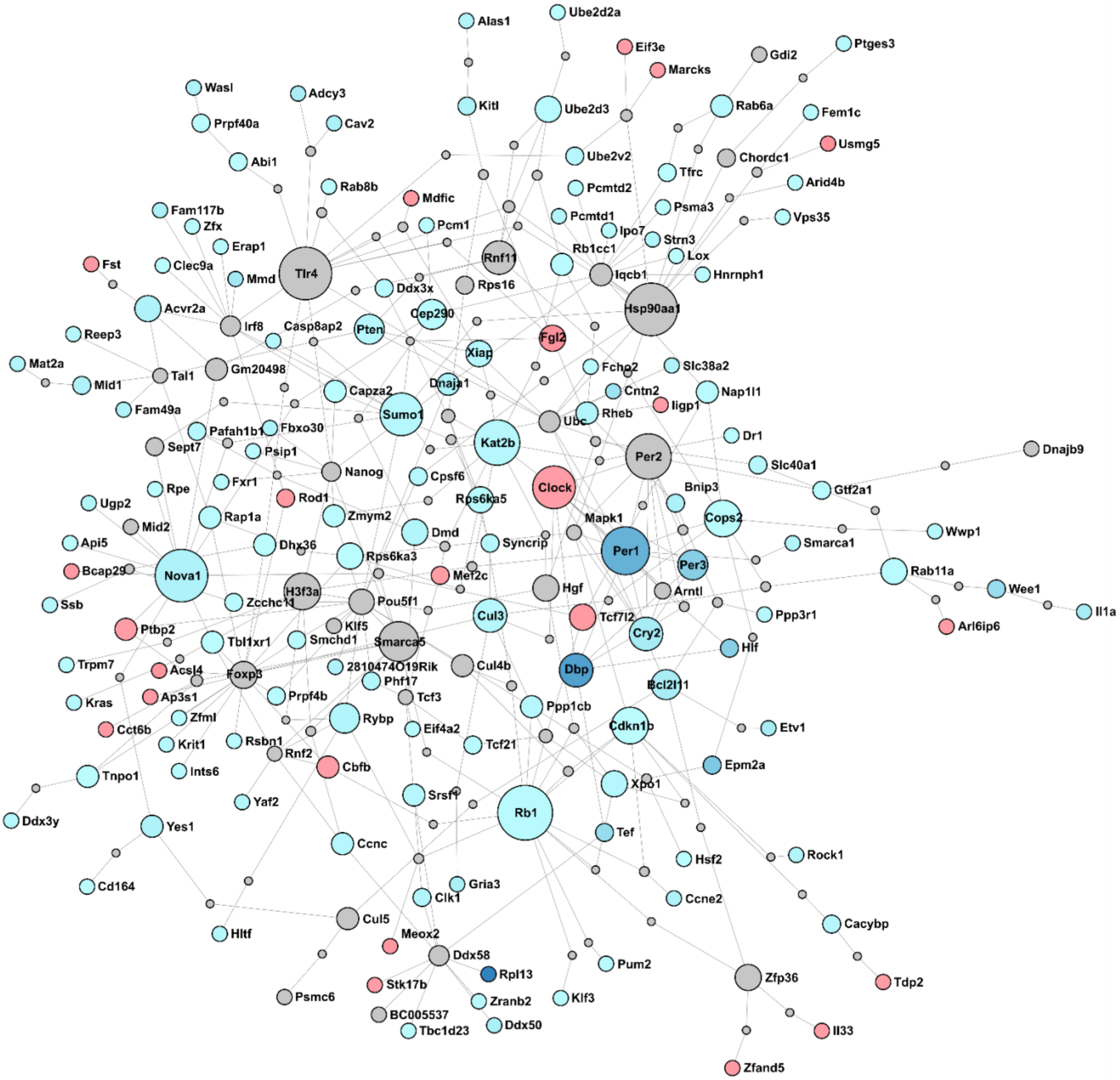

Figure S3. **Network representation of the PVG-specific lung module (yellow).** A minimum first-order protein-protein interaction network was created for the genes within the PVG-specific lung module associated with metabolism pathways using NetworkAnalyst and InnateDB. Genes were converted to mouse orthologs for compatibility with InnateDB. Grey nodes represent genes that are not contained within the module but are first-order neighbours. Red and blue colouring indicate increased and decreased expression respectively on day 9 relative to day 0.

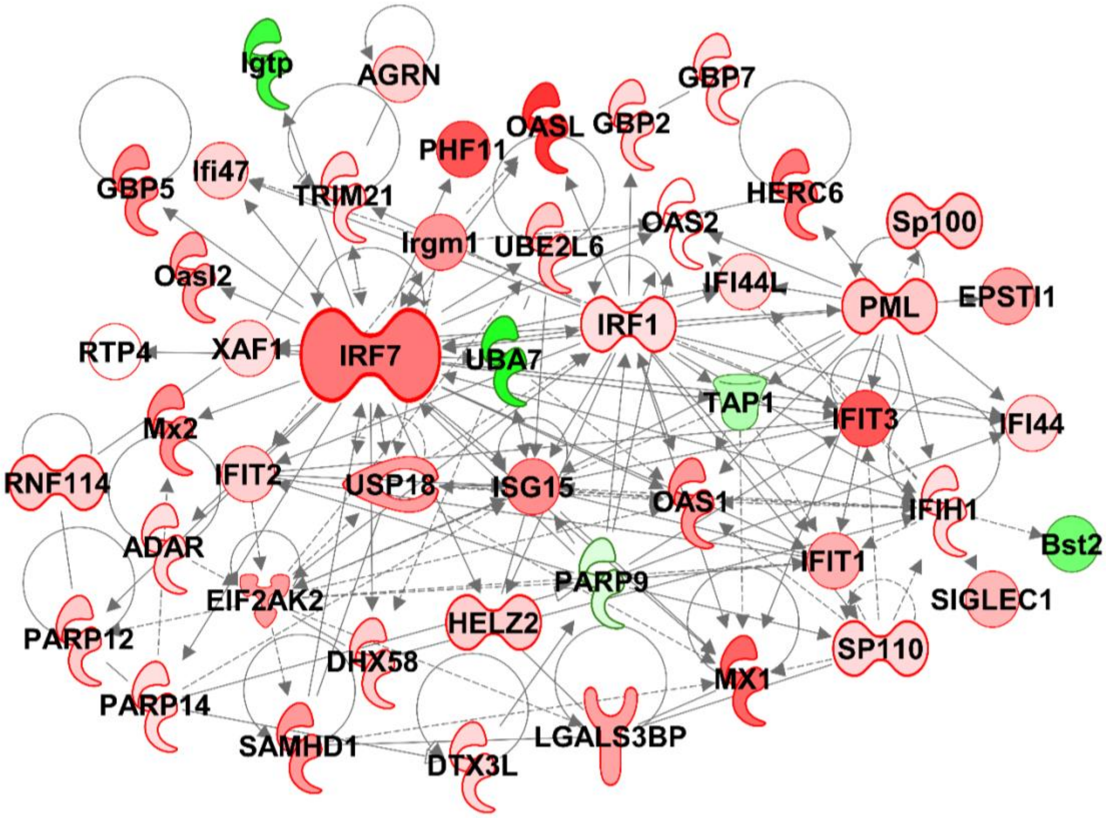

Figure S4. Network representation of the PVG-specific bone marrow module (salmon). A prior knowledge network was created for the genes within the PVG-specific bone marrow module associated with viral response/interferon pathways using Ingenuity Pathways Analysis software. Nodes are coloured based on the PVG response at day 9 (virus/allergen coexposure) where red and green indicate increased and decreased expression, respectively.

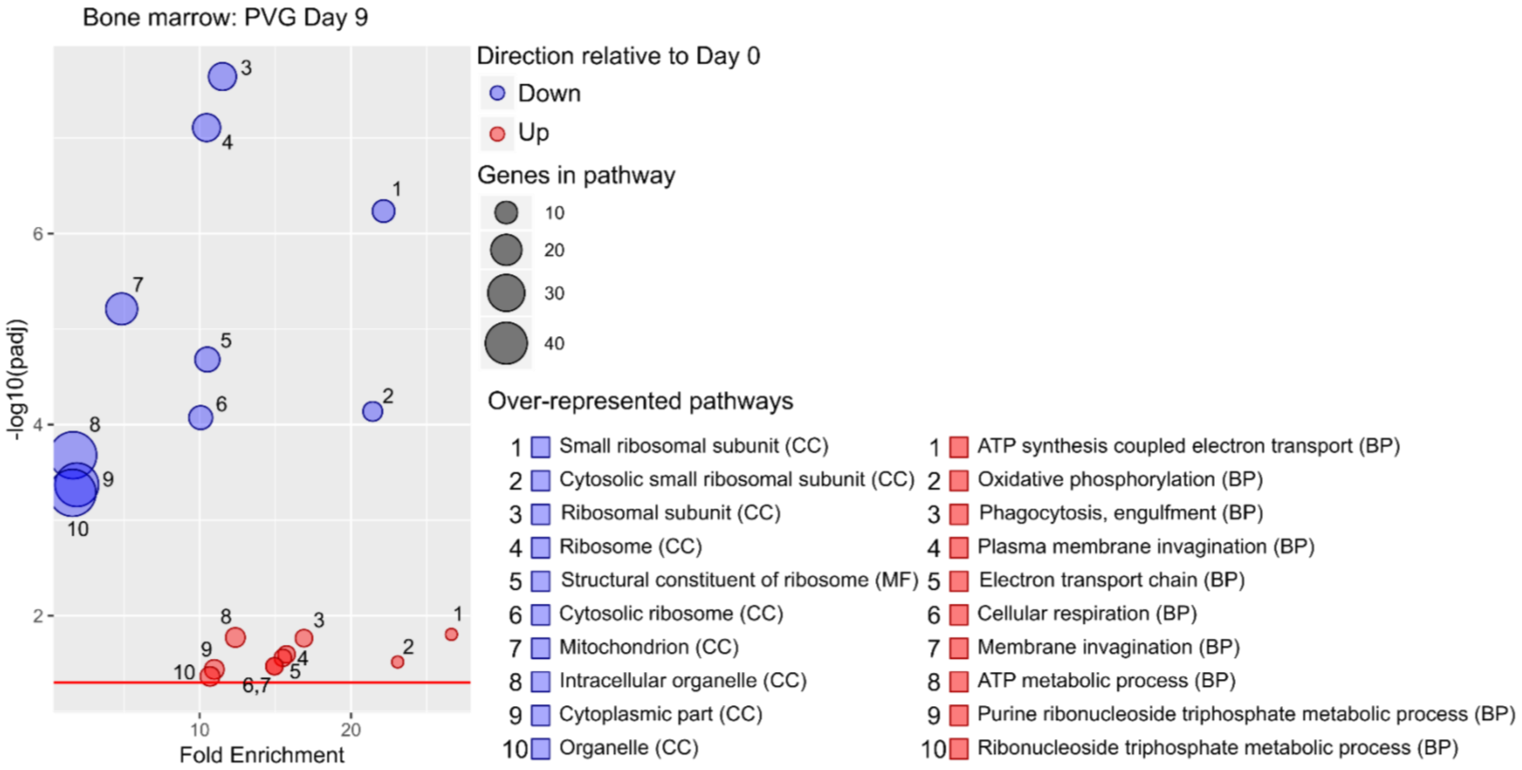

Figure S5. Over-represented biological pathways associated with virus- and allergen-induced gene expression changes in PVG bone marrow at day nine post-infection. Bubble plots show the over-represented biological pathways associated with the differentially expressed genes. Pathways with Bonferroni-corrected p-values <0.05 were considered significant (represented by the solid red line intersecting the y-axis).

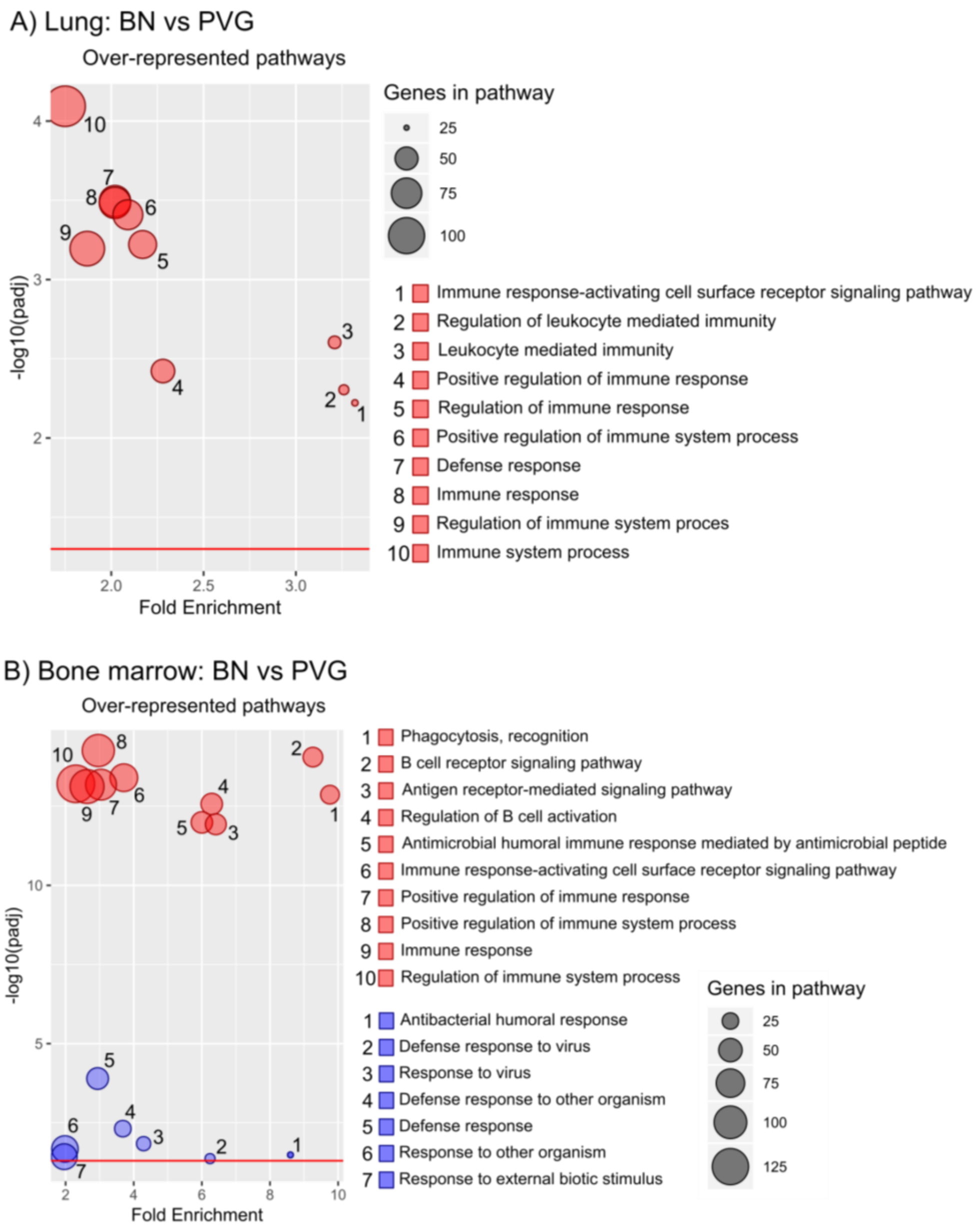

Figure S6. Over-represented biological pathways associated with baseline differences between BN and PVG rats. Bubble plots show the over-represented biological pathways associated with the differentially expressed genes between BN and PVG at baseline in lung (A), and bone marrow (B). Analysis was performed on genes with an absolute fold-change >1.5. Pathways with Bonferroni-corrected p-values <0.05 were considered significant (represented by the solid red line intersecting the y-axis).
